# A microfluidic platform towards automated multiplexed *in situ* sequencing

**DOI:** 10.1038/s41598-019-40026-6

**Published:** 2019-03-05

**Authors:** N. Maïno, T. Hauling, G. Cappi, N. Madaboosi, D. G. Dupouy, M. Nilsson

**Affiliations:** 1 Lunaphore Technologies SA, EPFL Innovation Park Building C, CH-1015 Lausanne, Switzerland; 2 Wolfson Institute for Biomedical Research, University College London, Gower Street, London, WC1E 6B United Kingdom; 3 Science for Life Laboratory, Department of Biochemistry and Biophysics, Stockholm University, Tomtebodavägen 23a, SE 171 65 Stockholm, Sweden

## Abstract

Advancements in multiplexed *in situ* RNA profiling techniques have given unprecedented insight into spatial organization of tissues by enabling single-molecule quantification and sub-micron localization of dozens to thousands of RNA species simultaneously in cells and entire tissue sections. However, the lack of automation of the associated complex experimental procedures represents a potential hurdle towards their routine use in laboratories. Here, we demonstrate an approach towards automated generation and sequencing of barcoded mRNA amplicons *in situ*, directly in fixed cells. This is achieved through adaptation of a microfluidic tool compatible with standard microscope slides and cover glasses. The adapted tool combines a programmable reagent delivery system with temperature controller and flow cell to perform established *in situ* sequencing protocols, comprising hybridization and ligation of gene-specific padlock probes, rolling circle amplification of the probes yielding barcoded amplicons and identification of amplicons through barcode sequencing. By adapting assay parameters (e.g. enzyme concentration and temperature), we achieve a near-identical performance in identifying mouse beta-actin transcripts, in comparison with the conventional manual protocol. The technically adapted assay features i) higher detection efficiency, ii) shorter protocol time, iii) lower consumption of oligonucleotide reagents but slightly more enzyme. Such an automated microfluidic tissue processor for *in situ sequencing* studies would greatly enhance its research potentials especially for cancer diagnostics, thus paving way to rapid and effective therapies.

## Introduction

Progress in sequencing technologies during the recent years has enabled to study the genomic and transcriptomic landscape of tissues, reaching down to even single cell level^1–7^. Methods such as single-cell RNAseq provide precise quantifications of RNA copy numbers but require cells dissociated from tissues as input material. Inherent to the technique, information about the spatial organization of the analyzed cells is lost. Molecular techniques to pinpoint the locations of individual RNAs have emerged, enabling the mapping of cell types and their interactions^7,8^, thus proving highly beneficial for understanding both biological mechanisms as well as clinically relevant processes such as disease progression. Such methods, regrouped under the name of spatial transcriptomic, achieve multiplexed transcript detection by combinatorial barcoding of single-stranded DNA probes that hybridize to target RNAs or cDNA thereby yielding target-specific signals^9–11^. Furthermore, many of these techniques require probe-target specific ligation event^12–14^ and subsequent amplification^12–17^. While devices for automation of bulk and single cell sequencing exist and are in routine use, spatial transcriptomic methods are technologically hampered by largely manual protocols. Instruments tailored to multiplexed in situ methods are missing or exist only as custom-built solutions for lab-specific microscopes. The complexity of the protocols that include multiple enzymatic steps, typically with different temperature requirements and buffer conditions, might explain the absence of automation.

**Figure 1:**
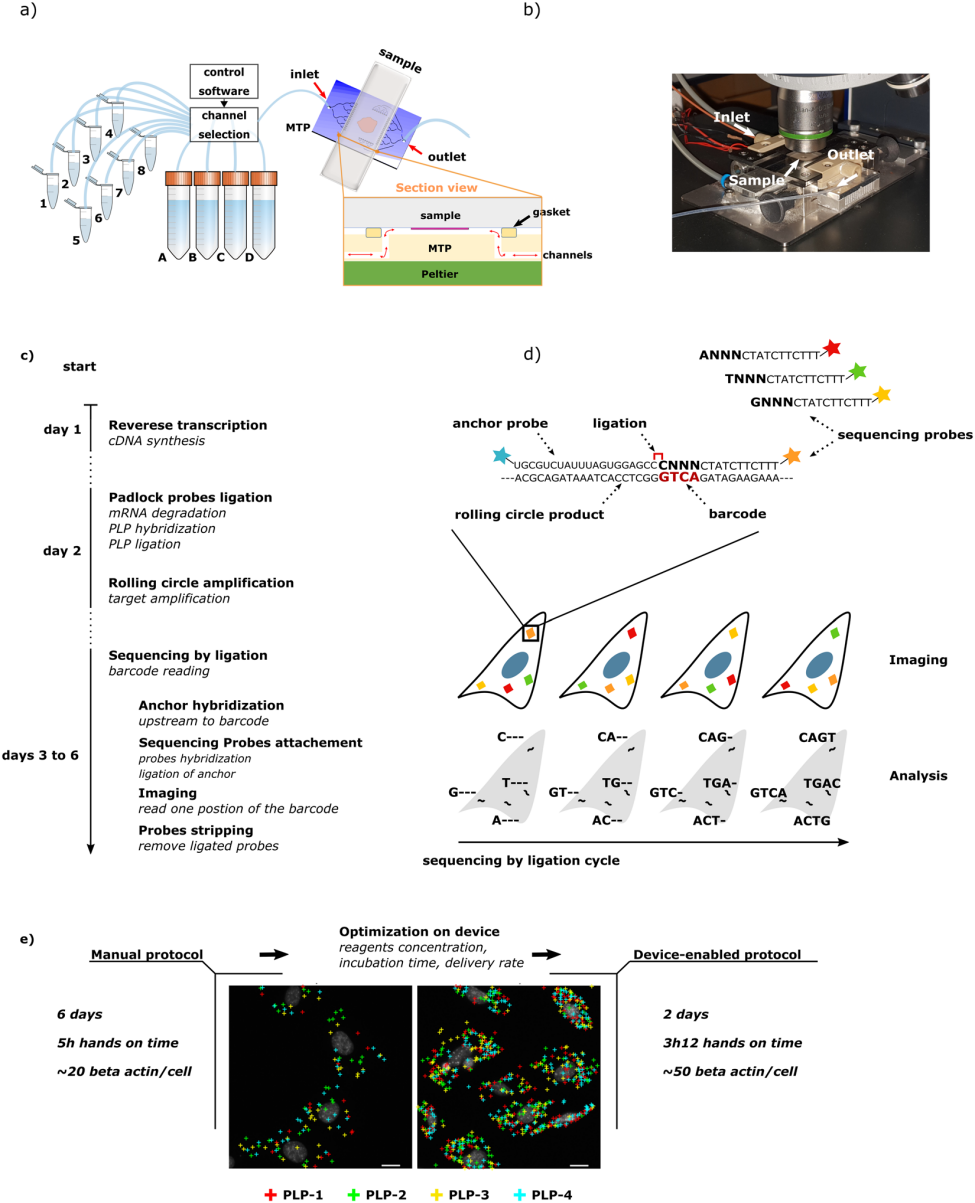
Assay scheme and description of the microfluidic tissue processor. (**a**) Working principle of the microfluidic technology. The microscope slide containing the sample is clamped to the MTP to form a reaction chamber of 17 × 17 × 0.1 mm^2^ where temperature is controlled by a Peltier element. Reagents are uniformly delivered in the reaction chamber thanks to the MTP micro-channels design. Reservoirs one to eight and A to D consisting of disposable eppendorf and falcon tubes respectively that were filed with the different reagents solution needed for the assay before mounting on the machine. Reagent delivery (e.g. polymerase mix, washing buffer) is controlled via software. The inset in figure shows the cross section of the clamped sample and MTP. (**b**) Picture of the sample processing unit on a microscope stage. (**c**) Scheme of the ISS assay with corresponding time schedule for a manually performed protocol. mRNA in the cells is reverse transcribed to cDNA. mRNA is then degraded to allow hybridization of molecularly barcoded PLPs to cDNA. Upon hybridization, a PLP circularize, bringing its two arms side by side on the target allowing them to be ligated. The formed circles are then amplified by RCA, producing RCPs which are nearly micron sized amplicons consisting of end-to-end repeats of the PLPs sequence. SBL of the RCPs barcodes finally allows to identify the original mRNA detected. The fluorescence signal is strongly amplified thanks to the high number of barcodes within RCPs. (**d**) Scheme of SBL cycles leading to a full RCPs barcode resolving. Information is read as fluorescence signal from sequencing probes during imaging, interpreted as nucleotide during analysis. Sequencing probes perfectly hybridize to RCPs except at the barcode positions where one fixed and three degenerate nucleotides allow to resolve this specific barcode’s nucleotide through preferential ligation of the matching sequencing probe to an upstream primer. Sequencing probes carry nucleotide-specific fluorophores. (**e**) Summary of the study with simple workflow of the optimization and comparison of the critical parameters of the assay (duration, hands on time and detection yield). Insets are corresponding images post-analysis obtained for application of the ISS assay to the detection of *Actb* in MEF cells using four PLPs (scale bars are 5 µm).

A commercially available microfluidic technology, based on a reversible reaction chamber formed at the interface with a glass slide (Fig. 1a,b), has been demonstrated to enable automated rapid immunohistochemical staining on tumor sections^18,19^. Recently, this technology has been applied to automation of fluorescence *in situ* hybridization (FISH)^20^. Similarly, it is envisaged that this platform has the features required to automate any of the above mentioned spatial transcriptomic assays  including in situ sequencing (ISS, Fig. 1c,d), which depends on multiple enzymatic steps with different temperature requirements and precisely adjusted buffer conditions.

In ISS, endogenous RNA in fixed tissue specimens is enzymatically converted to complementary DNA (cDNA) to which barcoded, single-stranded DNA probes (padlock probes - PLPs) hybridize^21^, thereby forming a circular structure upon the target strand. Typically, PLPs are designed such that their 5′ and 3′ termini hybridize directly adjacent to each other, separated only by a nick. This nick is closed in a ligation reaction catalyzed by a thermostable DNA ligase (derived from *Thermus thermophilus* DNA ligase), which ligates adjacent 3′ hydroxylated and 5′ phosphorylated ends. This process is highly specific since the ligase is sensitive to mismatches at the ligation site, ensuring that only completely hybridized PLPs are ligated. Primed by the target cDNA strand, the circular template is then locally amplified in a process known as rolling circle amplification (RCA)^22^ using DNA polymerase enzyme, thereby forming a typically nearly-micron sized single-stranded DNA cluster (rolling circle amplification product – RCP) that contains barcoded reverse-complementary copies of the original probe sequence. The PLP barcodes, rather than the original transcript sequences, are read directly in the specimen using sequencing-by-ligation (SBL) chemistry^23^. Samples are subjected to multiple, repeated rounds of base-specific labeling by fluorescent dyes, imaging, and dye-removal for the desired number of cycles until the original transcript is identified (Fig. 1d).

Up to date, only one PLP/RCA *in situ* assay has been adapted  to a robotic station^24^. This study brings emphasis at the microfluidic level for reagents delivery, mixing and washing, however, it does not encompass a multiplexing solution such as SBL. In the present study, we describe the adaptation of ISS chemistry  to Lunaphore Technology’s  microfluidic platform. This tool encompasses a reagents delivery system, a microfluidic tissue processor unit and a thermal controller (Fig. 1a,b) the whole being operated in a semi-automated fashion (i.e. manual reagents preparation followed by automated protocol execution) through a dedicated software. The aim of this study was to quantitatively measure to what degree of performance the device-enabled assay can be brought without set-up modification. This would effectively demonstrate that technology in affordable automated microfluidic has now caught up with the high complexity of methods such as ISS. This would in turn motivate the adaptation of other spatial transcriptomic assays to automated systems, and ultimately promotes their widespread use in the scientific community.

We demonstrate that optimization of individual assay modules allows to achieve at least the same performance as the manual protocol (Fig. 1e), but with reduced hands-on time through automation and overall assay speed. This proof-of-concept appeals to reader from the RNA-seq and more specifically from the spatial transcriptomic fields, showing that the tool used could enable other *in situ* hybridization/sequencing assays with expected benefit as described in this study. This work is also relevant to users of parallel methods such as ligation-based assays or isothermal amplification methods considering the information gathered during the step-by-step optimization of assay modules.

## Results

### General experimental design

In optimizing the complex protocol of ISS, the experimental conditions were chosen as simple as possible in order to remove as much complexity and variability as possible. Concretely, three constraints were placed over the experimental conditions: (i) the optimization was carried out using a cell line. This choice is justified by the intrinsically higher variability of gene expression in tissue compared to cell line^25^, and the fact that it is impossible to generate tissue sections with reproducible cellular and molecular content. Accordingly, mouse embryonic fibroblast (MEF) were used. (ii) only one gene transcript, the mouse beta-actin (*Actb*), was targeted despite the multiplexability of the method. This transcript was chosen over a criteria of stable expression level across cells and high transcript count per cell^26,27^. Nonetheless we motivate that the results obtained in this study with only one target would be reproducible in a multi-target assay. We designed four PLPs all targeting the same sub-sequence of the *Actb* transcript but having different barcodes thus generating four-plex ISS data. Furthermore, by using a four positions barcode, requiring four cycles of SBL, we can derive metrics to evaluate the performance of a 256-plex (4^4^) assay while being uninfluenced by gene expression variance between transcripts. To sum up, with our four PLPs with four base pair barcodes, we could imitate 16-, 64- and 256-plex assay conditions by submitting sample to two, three or four cycles of SBL. Ultimately, the PLPs’ barcodes were orthogonal. This is meant as an internal control for the SBL. Given that the PLPs have an equal sequence expect for their barcodes which doesn’t play a role in target hybridization, they have equal affinity to the target and as a consequence a non-uniform ligation of the sequencing probes would then be reflected by an increased detection of one (two) probe(s) over the others. (iii) each step of the protocol was optimized individually. Only the step of interest was performed on-chip (i.e. on the device) while the other steps in the procedure were performed manually.

Altogether these minimalistic constraints helped to reduce the noise and hence highlight the impact of single parameters changes (e.g. reagents concentration, incubation time, temperature) introduced in the protocol. Controls were systematically performed in the form of a manually performed assay in parallel (i.e. off-chip). The result of a given step optimization is then reported normalized to its control. This enabled a consistent way of reporting results throughout the whole optimization process regardless of reagents lot, cell batches or imaging conditions.

### Library preparation anchor hybridization optimization

The reverse transcription and formaldehyde fixation were always performed manually. Different variables were used to appreciate the performance of the assay depending on which step of the protocol was considered. For short, performance efficiency (PE) is used throughout this study as a synonymous phrasing of: step specific performance normalized to off-chip control. The performance of the library preparation, consisting of PLP hybridization and RCA only, was assessed by the RCP yield of the assay (mean RCP count per cell) and is reported normalized to the off-chip control. Given that we used four PLPs all targeting the same transcript (*Actb*) and that we pooled all the RCPs for counting, the RCP yield corresponds to the number of *Actb* transcripts detected per cell.

**Figure 2:**
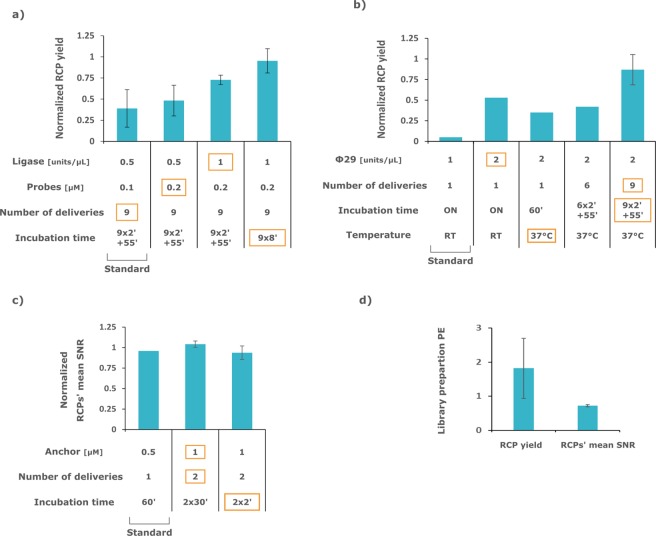
Optimization of library preparation and anchor primer hybridization on-chip. Optimization experiments, showing the RCP yield for (**a**) padlock probing, (**b**) RCA, and RCPs’ mean SNR for (**c**) anchor primer hybridization, all normalized to off-chip performance. Standard and optimized parameters correspond to the outermost left and right bars, respectively in (**a**–**c**). Incubation time parameter formulated as α × β′+ ʎ′ reads as α deliveries incubated β min each, followed by ʎ min final incubation. (**d**) Performance obtained by combining the optimized parameters derived from (**a**–**c**) on-chip, normalized to the manually performed control off-chip. For (**a**–**c**) all bars n = 2, biological replicates. For (**d**) n = 3 technical replicates. The red squares highlight the first occurence of a parameter’s final value, which was retained in the optimized, device-enabled, protocol.

As starting point for optimization, the standard protocol was first implemented on-chip. Because of the smaller dimensions of the MTP chamber compared to the off-chip hybridization chambers, the volumes dispensed on-chip were adapted to provide the same number of enzyme molecules per unit surface. By modifying one parameter at a time, the assay performance was gradually improved. A wide range of parameters was tested (Fig. S1), of which only the most potent ones were carried out for further experiments (Fig. 2a,b).

The next protocol step is the anchor primer hybridization. This anchor is fluorescently labeled and primes the subsequent SBL. To measure the performance of this step, the RCPs’ mean signal-to-noise ratio (SNR) was used and is again reported as normalized to its off-chip value. The optimization workflow for the individual assay modules is presented below.

#### PLP hybridization

The initial parameters on-chip corresponded to nine deliveries of 29 μL of 0.5 U/μl of AmpLigase and 0.1 μM of PLP (other co-factor and buffer concentrations are as described in the methods). Each delivery, effectively renewing the whole volume of the chamber, was incubated 2 min before a final incubation of 55 min. These parameters together yielded an initial 39% PE (Fig. 2a, here PE denotes normalized mean RCP yield). A modest improvement was first made by doubling the PLPs’ concentration, thus achieving 48% PE. Doubling the concentration of the ligase then boosted the PE to 72% and hence proved to be the most potent of all the tested parameters. At last, lengthening the incubation time of ligase from 2 to 8 min (in between chamber refill, final incubation reduced from 55 to 8 min) interestingly increased the PE to 95%. To summarize, the best parameters for this assay module were 9 deliveries incubated 8 min each with double the concentration of PLP and ligase.

#### RCA

The initial on-chip parameters corresponded to nine deliveries of 29 μL of 1 U/μl of phi29 polymerase incubated overnight at room temperature. The starting parameters were however chosen as a single delivery of 1 U/μl of phi29 for the economy of reagents but yielded only 5% PE (Fig. 2b). The total amount of phi29 delivered was hence gradually increased. Doubling phi29 concentration yielded 53% PE. At this point, the RCA incubation was reduced to 1 h at 37 °C which showed similar performance. Adding deliveries up to six and then nine resulted in achieving 42% and 87% PE. As for ligase in PLP hybridization module, increasing the enzyme concentration was the most efficient parameter in the RCA module. The final optimized parameters were 9 deliveries incubated 2 min each at 37 °C with doubled phi29 concentration and final incubation of 55 min at 37 °C.

#### Anchor hybridization

The initial on-chip parameters corresponded to nine deliveries of 29 μL with 0.5 μM of anchor primer and a total incubation time of 1 h. Again, we tried a minimalist approach by making a single reagents delivery incubating 1 h (Fig. 2c). The performance was as good as off-chip in term of RCPs’ mean SNR (96% PE). We hence aimed at shortening the incubation time. Doubling the concentration of anchor and adding a second delivery was evaluated to check for possible background increase. We observed no adverse effect as the normalized mean SNR (104% PE) was similar. We thus next decreased the incubation time of each delivery from 30 to 2 min and observed similar performance (93% PE). The final parameters were hence 2 deliveries with doubled anchor concentration incubated 2 min each.

To finally confirm the optimal parameters individually selected, PLP hybridization/ligation, RCA and anchor hybridization were run together on the device. The PE in terms of RCP yield and RCPs’ mean SNR were 181% and 72% respectively (Fig. 2d), thus comparable to the manual protocol. The total duration of the adapted protocol for the library preparation was five hours, equal to the manual protocol. The hands-on time was 50 min compared to 40 min for the manual protocol, slightly longer due to the setting up of the machine (Fig. S2a).

Additionally, the elute from the MTP to the waste was analyzed, looking for possible residual enzymatic activity. The elute was collected during the dispensing of the PLP ligation mix and we hence assessed ligation activity. After plausible circle formation and subsequent RCA catalyzed in solution, we counted the number of RCPs obtained by automated single molecule detection unit^28^. The residual ligation activity in the elute was below 1% of the positive control indicating that there was almost no active enzyme molecules lost to the waste (Fig. S3).

### SBL optimization

**Figure 3:**
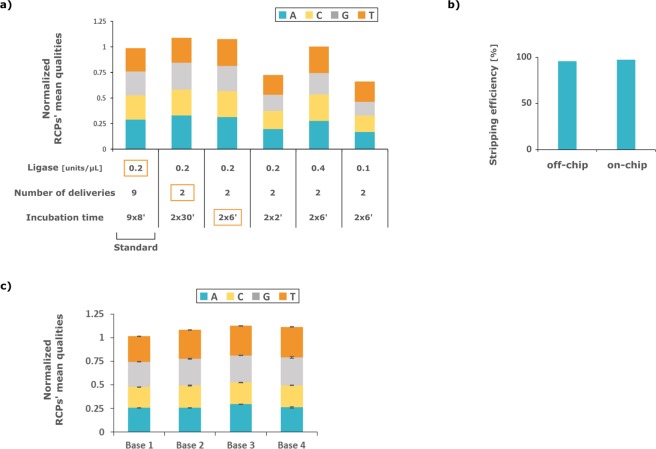
Optimization of the Sequencing-by-ligation on-chip. (**a**) Performance is assessed by averaging the RCPs’ quality within their corresponding sequencing probe groups (A, C, G and T), downscaling each one of them by a factor of four, stacking them and normalizing the result to the off-chip control. RCP quality is computed as the ratio of the assigned sequencing probe’s fluorescence intensity (i.e. strongest signal) to the sum of all sequencing probes’ fluorescence (all signals excluding anchor primer). Essentially, RCPs’ mean qualities stack being equal or higher than 1 denotes a base-calling as robust or better than control. The red squares highlight the first occurrence of a parameter’s final value, which was retained in the optimized, device-enabled, protocol. (**b**) Comparison of detection probes stripping efficiency between off-chip and on-chip. (**c**) RCPs’ mean qualities for the four bases of the barcode, normalized to off-chip. The steady value through different cycles indicates good stability of the RCPs. Error bars correspond to within-sample variation (n = 1).

In order to optimize the reagents mix and incubation time of the SBL, the library preparation was run manually in hybridization chamber for all the samples and the assay optimization was then performed on-chip, step by step as before. A single nucleotide of the RCP barcode was sequenced both off-chip and on-chip and the base calling was performed as in the original analysis pipeline^1^. Essentially, a nucleotide of a given RCP’s barcode is called based on the sequencing probe having the brightest signal of all. To appreciate the quality of a given cycle of base calling, the ratio of the brightest sequencing probe signal to the sum of all was computed for all RCPs individually. This measure is known as RCP quality in the original analysis pipeline. The RCPs’ qualities were average group-wise (i.e. A, G, C and T) and are here displayed as a stacked bar normalized to off-chip control and divided by four to rescale to 1 (Fig. 3a). In essence, higher bar correlates with more confidence in the base-calling. Correspondingly, stacked bar summing to one denotes base-calling as robust as the manually performed protocol. As explained above, because we used four PLPs of equal affinity for a common residue and having orthogonal barcodes, we expect to have an equal number of RCPs called in each base group (e.g. A, G, C and T), characterized by equal RCPs’ quality. In brief, the contribution of each nucleotide to the stack bars should be equal.

The initial parameters ran on-chip corresponded to nine deliveries of 29 μl with 0.2 U/μl of T4 Ligase incubated 8 min each (probes, formamide, co-factor and buffer concentrations as described in the methods). The initial parameters achieved 99% PE (Fig. 3a, here PE refers to normalized stacked rescaled RCP’s mean qualities). In an attempt to reduce the incubation time, deliveries dispensed only twice and incubated either 30 or 6 min each achieved a similar performance of >100% PE. For shorter incubation time, a large decrease in the PE was observable. Using higher or lower concentration of ligase was, respectively, equal or worse. Other parameters including higher detection probes concentration, less or more number of deliveries and higher temperature were investigated with either adverse or no effect (Fig. 2c). The final parameters selected were 2 deliveries with doubled concentration of ligase incubated 6 min each. The detection probes were stripped off on-chip with a single delivery of UNG enzyme at 0.1 U/μl (five times more concentrated than off-chip but effectively 1.6 times less total enzyme than manual protocol) followed by three 20% formamide washes incubated 1 min each at 65 °C. The overall stripping efficiency was as good as the off-chip results (Fig. 3b).

The parameters selected during the optimization of individual steps were merged into one continuous protocol that included the library preparation (starting after formaldehyde fixation) and sequencing on-chip of the four bases of the barcodes. Concretely, the experiment in Fig. 2d was continued with the optimized parameters for the SBL. The per-base RCPs’ mean qualities were computed to monitor the staining efficiency along four cycles of staining and stripping (Fig. 3c).

**Figure 4:**
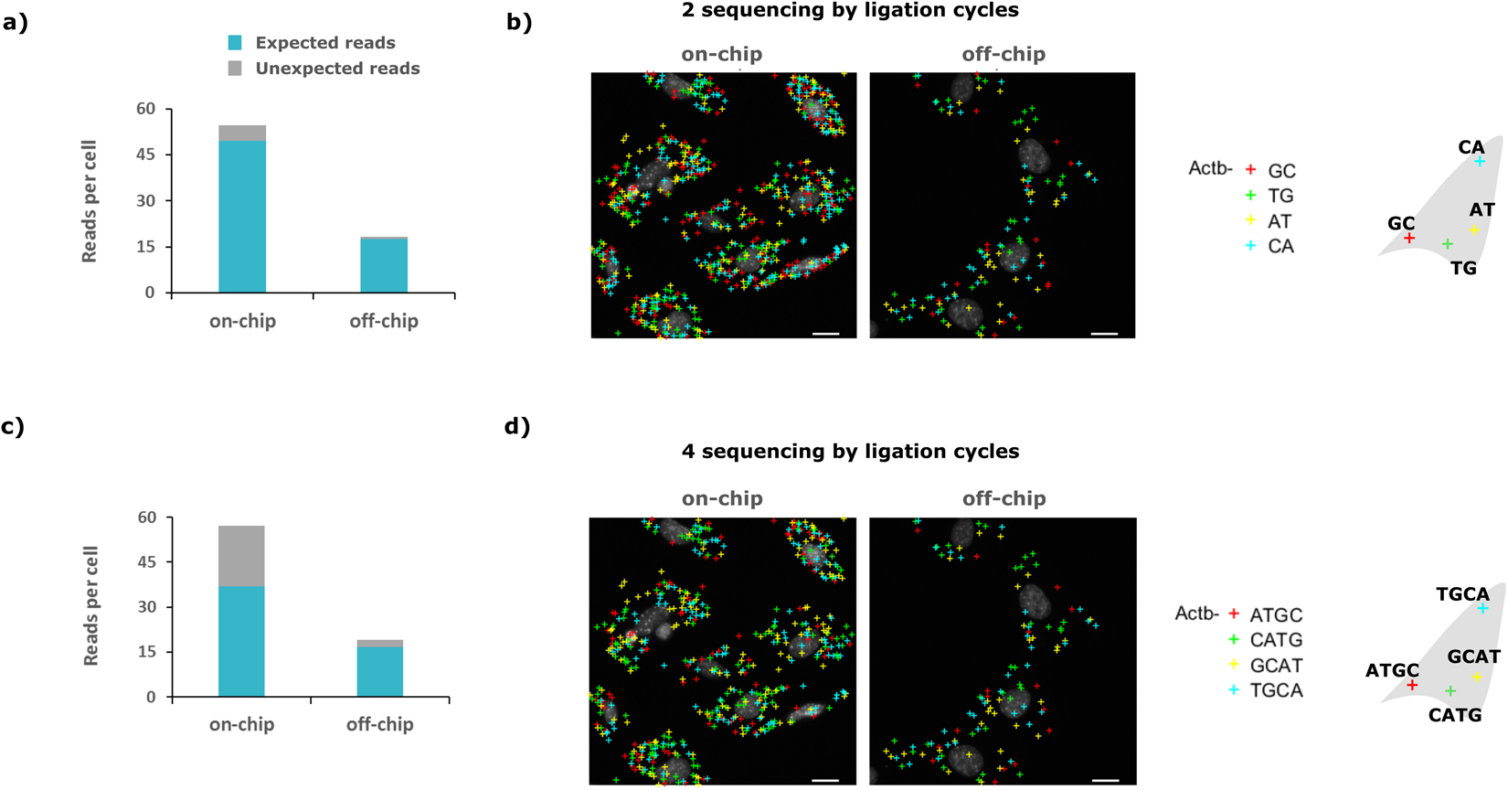
Read count pooled from the four padlock probes in the 16-plex and 256-plex assay conditions (**a**) *Actb* reads count per cell in the 16-plex assay conditions (i.e. after two hybridization cycles) for on-chip and off-chip protocol. Sequencing accuracy defined as the ratio of the expected to the sum of expected and unexpected reads, essentially the contribution of the blue bar to the total height of the stack bar, is 90% and 95% for on-chip and off-chip respectively. (**b**) Reads from (**a**) displayed over DAPI staining of cells’ nuclei. (**c**) *Actb* reads count per cell in the 256-plex assay conditions (i.e. after four hybridization cycles). Sequencing accuracy is 64% and 87% for on-chip and off-chip respectively. (**d**) Reads from (**c**) displayed over DAPI staining of cells’ nuclei. In (**a**,**b**) the reads from the four different padlock probes, all targeting the same epitope of *Actb*, are pooled together. Scale bars are 5 µm.

Once the whole barcode was read, the number of reads and sequencing accuracy were evaluated (Fig. 4). Here, read denotes the contiguous assembly of the bases called throughout the SBL cycles which is expected to be equal to one of the used PLPs’ barcode. Reads per cell is essentially the RCP yield after SBL and downstream analysis. In particular, a threshold is applied over the RCPs’ quality during analysis and RCPs which quality is below the threshold at any cycle of SBL are discarded. Again, reads per cell is obtained by pooling the reads originating from all four Actb PLPs and is thus synonymous of *Actb* transcripts per cell. ISS defines correct reads as expected while erroneous reads matching no PLPs’ barcode are defined as unexpected (expected: 4; unexpected: 252). Sequencing accuracy is then defined as the ratio of expected reads to the sum of both expected and unexpected reads. These parameters were evaluated considering either only two cycles (Fig. 4a) or all four cycles (Fig. 4b) of SBL. Two and four cycles of SBL technically allows to distinguish 16 (4^2^) and 256 (4^4^) PLPs in the assay respectively and hence enable 16- and 256-plex assay. The on-chip protocol achieved a higher detection efficiency, detecting 49.6 expected reads per cell against 17.5 off-chip in the 16-plex assay conditions (Fig. 4a), 36.8 expected reads per cell against 16.7 off-chip in the 256-plex assay conditions (Fig. 4b). The sequencing accuracy followed an opposite trend: on-chip protocol resulted in 90% accuracy compared to 95% off-chip (Fig. 4a) and 64% compared to 87% off-chip (Fig. 4b) in the 16-plex and 256-plex assay conditions respectively. The whole device-enabled protocol was achieved in two days compared to three days off-chip working time, which corresponds to two and six days in practice, respectively. In particular, the whole barcode reading was achieved in five hours on-chip instead of thirteen and a half hours off-chip (Fig. 2b).

## Discussion

Here, we demonstrate the adaptation of the ISS  to a microfluidic platform towards assay automation. Various assay parameters including incubation time, temperature, concentration and delivery rate of enzymatic or nucleic acid components were systematically tested (Figs 2 and 3). The automation brought by the liquid dispensing system and thermal controller allowed to reduce the total assay duration from 18 hours 37 minutes to 10 h and hands-on time from 4 hours 38 minutes to 3 hours 12 minutes (Fig. S2c). Notably, the total time for the SBL was reduced from 13 hours 37 minutes to 5 hours. In practice, the assay was achieved in six days with the manual protocol against two with the device. The consumption of reagents, despite being 1.5 times higher for enzymes, was reduced 2.5 times for oligonucleotides (Fig. S4), which remains the major scaling cost for multiplexed assays.

The individual optimizations of the library preparation modules were successful for all PLP ligation, RCA and anchor primer hybridization with PE of 95%, 87% and 93% respectively (Fig. 2a–c). Additionally, relevant results can be drawn from each of the individual module optimization. Considering the PLPs hybridization/ligation module, it is interesting that longer incubation in between deliveries rather than after has a positive effect (Fig. 2a), this over a time scale that is largely past the 150 seconds necessary for a 77 kDa ligase to diffuse through 100 µm^29^. This finding is of interest for other *in situ* ligation-based assays^13,14,17^. Similarly, the potency of using a higher number of polymerase deliveries for higher yield is of particular interest for isothermal amplification *in situ*^14,17,24^. This observation is coherent with the fact that phi29 has a half-life of 18 min at 30 °C ^30^ and motivates the use of a shallow microfluidic chamber. For a polymerase having diffusion coefficient of 70 µm^2^/s, we estimated that in the manual protocol set up almost two third of enzyme molecules in solution takes longer to diffuse to the sample than their half-life. Finally, the stark reduction of hybridization time is of interest for all *in situ* hybridization-based methods^9^. We again attribute this results to the shallowness of the MTP chamber that allows to efficiently replenish the depletion layer above the sample.

When the whole library preparation was performed on-chip, the RCP yield of the device-enabled assay, interpreted as the detection efficiency, was at least as good as the manual one, detecting 58.9 *Actb* transcripts per MEF cells compared to 38.6 respectively (corresponding to 181% PE, Fig. 2d, for normalized average computation see *methods*). On the other hand, the mean SNR of the anchor primer was lower on the device-enabled protocol, 8, compared to the manual protocol, 11 (corresponding to 72% PE, Fig. 2c). The SNR of the anchor primer is as relevant in ISS as it is in smFISH given that it is used during analysis to detect RCPs, resolving their position and defining the surface area over which sequencing probes fluorescence will be interrogated during analysis. Still, we appreciate the SNR on-chip as acceptable. We reach this conclusion given that the RCP yield is higher on-chip than off-chip, indicating that an SNR of 8 is enough to resolve RCP in an efficient enough scheme that allows the on-chip detection efficiency (RCP yield) to outperform the manual protocol, seemingly owing to the benefit brought by the optimization of the other assay modules. It is noteworthy that if necessary, the tool used in this study is amenable to high degree of performance for hybridization-based assays such as FISH, in particular in term of SNR, as was demonstrated in a recent study^20^.

The platform performed as well for the adaptation of the SBL module as it did for the library preparation, achieving 107% PE which here denotes mean RCPs’ qualities (Fig. 3a), again similar to what is achieved in the manual protocol. The time needed for hybridization and ligation of the sequencing probes was decreased by one hour, consistent with the results obtained for the anchor primer hybridization (Fig. 2c). Additionally, the amplicons (RCPs) could be restained efficiently over four successive cycle of SBL owing to the near perfect stripping of the sequencing probes achieved (Fig. 3b,c). When the whole barcode was sequenced the number of reads per cell post-analysis was 38.6 on-chip compared to 16.7 off-chip (Fig. 4a). As explained above, the RCPs originating from the four different PLPs could already be distinguished after two cycles of SBL. In this case 49.6 reads per cell on-chip compared to 17.5 off-chip were obtained (Fig. 4b). Correspondingly, the device-enabled protocol achieved good sequencing accuracy over two cycles of SBL, which can enable up to 16-plex detection, with 90% of reads being correct compared to 95% for the manual protocol (Fig. 4a). Over four cycles of SBL which corresponds to 256-plex assay conditions, accuracy dropped to 64% of correct reads compared to 87% off-chip (Fig. 4b). We appreciate the performance in the 256-plex conditions as still satisfactory given that the correct read per cell count is still in favor of the device. The orthogonal PLP barcodes allowed to verify that there was no bias towards the ligation or fluorescence of one sequencing probe over the other. This issue that can arise from the imaging setup (e.g. fluorophore, filters, camera) or the probes themselves (e.g. thermal or chemical degradation, lot) was qualitatively ruled out by the stack bars having equal contribution from each nucleotide for all four cycles of hybridization (Fig. 3c). The overall good performance and dramatic time shortage of the SBL module on the device is appealing for other SBL based method^14,17^.

Unlike a previous work that described automation of another spatially resolved transcriptomic assay^31^, in this work the cells were not dissociated after library preparation. This would enable to preserve the native structure of a tissue sample processed using this platform throughout the whole protocol. While automated system for hybridization assays are commonplace, corresponding affordable technical units for tissue preparation involving complex staining protocols such as ISS does not exist. Thus, the implementation of both library preparation and sequencing modules on the same platform, all involving complex enzymatic reactions, is the main achievement of this study. Moreover, the tool has the potential to enable imaging directly on the device without modification, while the assay is being performed. In fact, the MTP is fully detachable, small and light enough to fit on a microscope stage and with an opening in the back of the microscope slide (Fig. 1b). This would easily allow to follow enzymatic reactions with imaging on-chip of coverslips seeded cells or fixed tissues, effectively turning any fluorescence microscope capable of reading four fluorophores into an *in situ* sequencer. Accordingly, in addition to be an automated solution for complex enzyme-based assays, the tool could also solve the problem of imaging set-up thereby meeting a critical need in the field. In fact, there has been no previous description of a commercially available, stand-alone, automated tool with such feature and consequently, these methods have to a large extent remained in the hands of the persons who devised them. This study hence constitutes a promising advance for the field of spatial transcriptomics by proposing a stand-alone, automated staining device for any microscope equipment.

In conclusion, we have demonstrated the feasibility of adapting a complex and sensitive protocol on an affordable semi-automated microfluidic platform. This proof-of-concept outlines an efficient framework for assay optimization that, with the platform used in this study, could bring ISS and other spatially resolved transcriptomic assays closer to wide scale operation and envisaged wholesome integration in the laboratories.

## Methods

### Reagents

DMEM cell culture medium was purchased from Gibco. Fetal bovine serum, 2 mM l-glutamine and PEST, trypsin-EDTA, bovine serum albumin, 37% formaldehyde, formamide, glycerol, sodium citrate Buffer were purchased from Sigma. Secure-Seal hybridization chambers were purchased from Invitrogen. Superfrost Plus slides and Uracil-DNA glycosylase were purchased from Thermo Fischer Scientific. All oligonucleotides were purchased from Integrated DNA Technologies. Petri dishes were purchased from Corning. TranscriptMe, RiboLock RNase Inhibitor, RNase H, T4 ligase, deoxyribonucleotide triphosphate, adenosine triphosphate were ordered from DNA Gdansk. Ampligase was purchased from Epicentre, Illumina. phi29 DNA polymerase was purchased from Monserrate. DAPI was purchased from Biotum.

### MTP for automated ISS protocol

The automated microfluidic device from Lunaphore’s technology is employed for the automation and optimization of the ISS protocol on MEFs. The machine is composed of a reagents handling/pressure controllers unit and a freely movable MTP having dimension of 127.7 × 85.5 × 22.8 mm and weight of 460 g. A microscope slide containing the cell sample is loaded and then clamped to the MTP unit to form a closed chamber of 17 × 17 mm^2^ (Fig. 1a). The sealing relies on a polydimethylsiloxane (PDMS) gasket that defines the chamber thickness to 100 µm. Microfluidic inlet and outlet channels ensure the uniform delivery of the reagents, selected from a pool of eight 1.5 mL vials and four 50 mL falcons available. Under the MTP, a Peltier element (Laird Technologies SH10, 125, 05, L1, W4.5, USA), resistive thermometer (Heraeus PT100 FK222, Germany) and a controller (BelektroniG OEM-K20, Germany) enable to control the temperature inside the chamber (Fig. 1a,b). The operation of the machine is fully controlled by a dedicated software that allows the automated run of a protocol without any intervention from the operator. The MTP has an opening making the back of the sample visible. Upright fluorescence microscopy is readily achievable with coverslip seeded samples. In this study, however, the samples were always unmounted and prepared for imaging as in the manual protocol as described below.

### Cell culture

MEF cells (obtained from ATCC) were cultured in dulbecco’s modified eagle’s medium without phenol red and l-glutamine, supplemented with 10% FBS, 2 mM l-glutamine and 1× PEST and were incubated at 37 °C, 5% CO_2_.

To prepare cell samples, confluent cell lines were treated with 0.25% (w/v) trypsin-EDTA and resuspended in culture medium to a final concentration of 15,000 cells per milliliter. Resuspended cells were then seeded on five Superfrost Plus slides placed in a 150 mm × 25 mm Petri dish, and culture medium was added to a final volume of 22 ml. Cells were incubated in the same previous conditions 12–24 h before fixation. Fixation was performed in 4% (w/v) formaldehyde in DEPC treated phosphate-buffered saline (DEPC-PBS) for 15 min at RT after removal of the culture medium and washed once in PBS. After fixation, slides were washed once in DEPC-treated PBS and dehydrated in an ethanol series of 70%, 85% and 100% for 3 min each.

### ISS protocol

Before optimization, the protocol was identical to the previously described one^12^. Briefly, mRNA was reverse transcribed to cDNA by adding 5 μM of random decamer primers, 20 U/μl of TranscriptMe, 1 mM deoxyribonucleotide triphosphate (dNTPs), 0.2 μg/μl bovine serum albumin (BSA) and 1 U/μl RiboLock RNase Inhibitor in diethyl pyrocarbonate treated water (DEPC H_2_O). The reaction was incubated at 37 °C overnight (ON). Fixation of cDNA was allowed for 1 h at room temperature (RT). *Actb* was selected as target for its cellular abundance. Four PLPs targeting the same mRNA region were designed with barcodes such that all four bases were detected during each sequencing cycle. Degradation of RNA, and hybridization and ligation of PLPs were carried out for 30 min at 37 °C and 1 h at 45 °C respectively using a single reaction mix of 1× Ampligase buffer, 25 nM of each PLP, 0.5 U/μl Ampligase, 0.4 U/μl RNase H, 50 mM KCl and 20% formamide in DEPC H_2_O. RCA was performed either overnight at RT or for 1 h at 37 °C, as specified in text, with 1 U/μl phi29 polymerase, 1× Ampligase buffer, 0.25 mM dNTPs, 0.2 μg/μl BSA and 5% glycerol in DEPC H_2_O. Anchor hybridization was allowed for 1 h at RT with 500 nM of uracil-containing anchor primer, 0.5 μg/mL DAPI, 2x saline sodium citrate buffer (SSC) and 20% formamide in deionized water (dH_2_O). Hybridization and ligation of sequencing probes was performed for 1 h at RT with 0.1 μM (for A, C and G bases) and 0.3 μM (base T) of probes, 1× T4 ligase buffer, 1 mM adenosine triphosphate (ATP) and 0.1 U/μl of T4 ligase in DEPC H_2_O. Uracil-DNA glycosylase (UNG) treatment was performed in 0.02 U/μl UNG and 0.2 μg/μl BSA in a homemade buffer (1 × 200 mM Tris-HCl, 100 mM EDTA, 100 mM NaCl, pH 8) in DEPC H_2_O. Finally, 65% Formamide stripping wash was applied three times for one minute each. DEPC-PBS supplemented with 0.05% Tween-20 (DEPC-PBS-T) washes and ethanol series were performed intermittently during the protocol as originally described. Slides were mounted with Invitrogen slow fade mounting medium. Images were acquired using a motorized epifluorescence microscope (Axio Imager.Z2, Zeiss), equipped with a white light metal halide source (HXP120), a digital camera (Hamamatsu Flash4) and single bandpass excitation/emission filters (SP102v2, SP103v2, SP104v2, 49007 (all Chroma) & 49DAPI, 38HE (Zeiss) to separate DAPI, Cy7, Cy3, Cy5 Texas Red and AlexaFluor 488. Under-exposure and saturation were avoided by carefully adjusting exposure times, slide per slide. Three to seven field of views were acquired per condition to get at least 100 cells per condition. All raw images contributing to the displayed data are available on demand.

When performed off-chip (i.e. manually), all reactions were performed in 9 mm of diameter × 0.8 mm of height Secure-Seal hybridization chambers. The chamber was removed during the ethanol series before imaging.

When done on-chip (i.e. on the device), the protocol was carried out by loading the microscope slide with the cells’ sample on the automated microfluidic device. The optimization of the ISS protocol included the concentration of reagents, reagents mix composition, presence of blocking solutions, incubation time of each reagent, dispensed volume and flow rate of each reagent, and influence of temperature. Reagent mixes were loaded into the vials and kept on ice during the execution of the protocol. Ethanol series dehydration was used to remove and remount the hybridization chamber when needed. The first step of the protocol on-chip was always a dispense of DEPC-PBS-T to rehydrate the samples. The images were acquired off-chip after mounting the slides with a cover slip.

### Residual enzymatic activity in microfluidic chip elute assessment

The elute from the MTP was collected during the delivery of the ligation mix and potential ligation activity was tested. The elute was diluted four times in a solution of dH_2_O with 0.2 µg/µl BSA, 10x AmpLigase buffer, PLPs (10 pM) and a complementary synthetic target (50 pM). As positive control, a fresh ligation mix (as in the standard ISS protocol less the PLPs and RNaseH) was similarly diluted four times in the same solution. As negative control, the elute was diluted four times but PLPs/synthetic targets were omitted. These mixes were incubated for 40 min at 45 °C to allow for eventual ligation activity. RCA was then performed in the same solution for 1 h at 37 °C and finally, the detection oligonucleotides were added. The number of RCPs were counted using an Aquila 400 (Q-Linea) digital fluorescent object counter^28^.

### Image analysis

Image analysis followed the original pipeline^1^. The parameters used to appreciate the different assay modules performance were computed as follows. (i) The RCP yield was computed as the number of RCPs per cells and meant as a general measure of the assay performance for the library preparation. (ii) The RCPs’ mean SNR was assessed from the fluorescence of the anchor primer only, computed as the ratio of the fluorescence intensity of each RCP to its surrounding within an eight pixels region. (iii) The RCP’s quality was defined as the maximum signal (i.e., intensity of the brightest sequencing probe) divided by the sum of all the signals. Based on the strongest fluorescing sequencing probe, RCPs’ qualities are assigned to the corresponding probe group during base-calling (A, C, G or T). These RCP’s qualities are computed for each hybridization step. (iv) The number of expected/unexpected reads per cell was computed by dividing the number of correct reads (reads matching one of the PLPs barcode) or erroneous reads (reads not matching any barcode) respectively by the number of cell. (v) Sequencing accuracy, computed as the ratio of expected reads to both expected and unexpected reads. We evaluated the sequencing accuracy after applying a quality threshold, over the RCP’s quality as in the original analysis pipeline, yielding a sequencing accuracy of 95% for the manually performed controls.

## Supplementary information

Supplementary information
