# Supplementary material for: A microfluidic platform towards automated multiplexed in situ sequencing
Source: Sci Rep. 2019 Mar 5;9:3542. doi: 10.1038/s41598-019-40026-6 (PMC6401021; doi:10.1038/s41598-019-40026-6)
Supplement: Supplementary file 1 — Supplementary information [file 41598_2019_40026_MOESM1_ESM.pdf]

i. Title

A microfluidic platform towards automated multiplexed *in situ* sequencing.

ii. Authors and affiliation

Maïno[1,3+], N., Hauling[2,3+], T., Cappi[1], G., Madaboosi[3], N., Dupouy[1], D., Nilsson[3*], M.

1. Lunaphore Technologies SA, EPFL Innovation Park Building C, CH-1015 Lausanne, Switzerland
2. Wolfson Institute for Biomedical Research, University College London, Gower Street, London WC1E 6B, United Kingdom
3. Science for Life Laboratory, Department of Biochemistry and Biophysics, Stockholm University, Tomtebodavägen 23a, SE 171 65, Sweden

+ Authors contributed equally to this work

* Corresponding author: mats.nilsson@scilifelab.se

iii. Supplementary information

a)

RCA: Incubation Temperature	Overnight Room temperature	6 hr RT	ON RT	1 hr 37°C	1 hr 37°C	1 hr 37°C	3/2/1 hr 37°C	1 hr 37°C
RCA: Enzyme	phi29: 1 U/uL	28.9uL	28.9uL - 2x (doubled phi29 concentration)	28.9uL - Enzyme/Substrate ratio	28.9uL -2x	28.9uL - 4x/2x/1x	28.9uL - 2x	28.9uL - 2x
RCA: buffer/reagents			Amp buffer		various Glycerol /BSA concentrations			dNTPs delivery every 5mn
Comments	% RCP yield is relative to the manually the performed control	<5% RCP yield	first progress	1:1 optimal	no effect	2x optimal	no benefit of longer incubation	no benefit

RCA: Incubation Temperature	Overnight Room temperature	2/1 hr 37°C	1 hr 37°C	1 hr 37°C	6x2mn + 55mn 37°C	6x2mn + 55mn 37/45°C	9x2mn + 55mn 37°C
RCA: Enzyme	phi29: 1 U/uL	28.9uL - 2x	28.9uL - 2x	28.9uL - 2x	28.9uL - 2x or 6x28.9uL- 1x	6x28.9uL - 1x	9x28.9uL - 2x
RCA: buffer/reagents		dNTPs delivery every 30mn	lower cell density - bigger reaction chamber	lower BSA concentration			
Comments	% RCP yield is relative to the manually the performed control	no benefit	cell to cell variability decreased	modest effect	50% RCP yield	no benefit	0.87% RCP yield

b)

RNAse/Padlock: Incubation Temperature	30mn -37°C 1h - 45°C	9x2mn + 20 mn - 37°C 9x2mn + 55mn - 45°C	9x2mn + 20 mn - 37°C 9x2mn + 55mn - 45°C	9x2mn + 20 mn - 37°C 9x2mn + 55mn - 45°C	9x2mn + 20 mn - 37°C 9x2mn + 55mn - 45°C	9x2mn + 20 mn - 37°C 9x2mn + 55mn - 45°C	3x10mn -37°C 9x2mn + 55mn - 45°C	9x2mn + 20 mn - 37°C 9x8mn - 45°C
RNAse/Padlock: Enzyme	AmpLigase: 0.5 U/uL RNAse: 0.4 U/uL	standard - 9x25 uL			2x AmpLigase	2x AmpLigase	2x AmpLigase RNAse: 3x28.9 uL	2x AmpLigase
RNAse/Padlock: buffer/reagents			2x Probes (doubled PLPs concentration)	4x Probes	2x Probes	4x Probes	2x Probes	2x Probes
Comments	% RCP yield is relative to the manually the performed control	56 % RCP yield	48% RCP yield	37% RCP yield	73% RCP yield	73% RCP yield	79% RCP yield	97% RCP yield

c)

SBL: Incubation Temperature	60 mn - RT	9x8 mn	9x2 mn	2x30 mn	1x30 mn	2x15 mn	2x15 mn - 37 °C
SBL: Enzyme	T4Ligase 0.2 U/uL						
SBL: Oligo	Detection probes: A,C,G : 0.1 uM T : 0.3 uM						
Comments	% of summed RCP's mean quality relative to the manually performed control	99 % RCP's mean quality	80 % RCP's mean quality	109 % RCP's mean quality	59 % RCP's mean quality	107 % RCP's mean quality	109 % RCP's mean quality

SBL: Incubation Temperature	60 mn - RT	2x6 mn	2x2mn	2x6 mn	2x6 mn	2x6 mn
SBL: Enzyme	T4Ligase 0.2 U/uL				0.5x Ligase (halved ligase concentration)	2x Ligase
SBL: Oligo	Detection probes: A,C,G : 0.1 uM T : 0.3 uM			2x Probes (all detection probes concetration doubled)		
Comments	% of summed RCP's mean quality relative to the manually performed control	107% RCP yield	72 % RCP's mean quality	110 % RCP's mean quality	66 % RCP's mean quality	103 % RCP's mean quality

Supplementary table 1: Parameters considered during optimization of (a) RCA, (b) PLPs hybridization and (c) SBL. Texts in red denotes the parameter of interest (e.g. changed) for this experiment.

a)

Library preparation

	incubation time	hands-on time	total
on-chip	4h30	50'	5h
off-chip	4h30	40'	5h

b)

Sequencing by ligation

	incubation time	hands-on time	imaging	total
on-chip	1h53	2h22	45'	5h
off-chip	8h54	3h58	45'	13h37

c)

Total

	incubation time total	hands-on time total	grand total
on-chip	6h23	3h12	10h
off-chip	13h24	4h38	18h37

Supplementary table 2: Assay incubation hands-on and total time for a) library preparation, b) sequencing-by-ligation and c) overall. The hands-on time for the library preparation is not cumulative with the incubation time as some reagents preparation are performed over the incubation times.

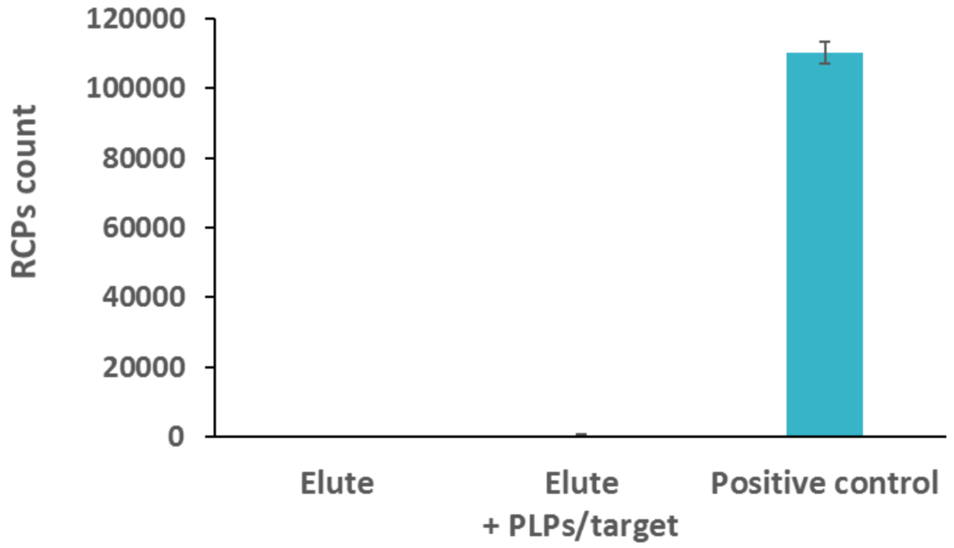

Supplementary figure 3: Analysis of elute during dispensing of the PLP ligation mix. A mix containing PLPs and a synthetic target was either added or omitted (negative control, outer most left bar). Additionally, a fresh mix containing Ligase (as in material and methods) together with PLPs/target was used a positive control (outer most right bar). Hypothetic ligation was allowed in solution followed by RCA and labeling. RCPs were counted using an automated fluorescent object counter. For each conditions n=3 technical replicates.

Enzymes		ON			OFF		
	C0	C_on	#delivery	amount	C_off	#delivery	amount
RNAseH	0.4	0.4	9	3.6	0.4	8	3.2
Ligase	0.5	1	9	9	0.5	8	4
phi29	1	2	9	18	1	8	8
T4	0.2	0.2	8	1.6	0.2	32	6.4
UNG	0.02	0.1	3	0.3	0.02	24	0.48
				32.5			22.08
			Ratio:	1.47192029			

Nucleic acids		ON			OFF		
	C0	C_on	#delivery	amount	C_off	#delivery	amount
PLP	0.1	0.2	9	1.8	0.1	8	0.8
Anchor	0.5	1	8	8	0.5	32	16
Seq-Probes	0.6	0.6	8	4.8	0.6	32	19.2
				14.6			36
			Ratio:	0.40555556			

Supplementary table 4: Comparison of enzymes and nucleic acids components consumption between on- and off-chip for a full assay (library preparation and 4 base barcodes reading). *C0* is the standard protocol component's concentration. *C_on* and *C_off* are the concentrations used in the optimized and standard protocol respectively. In the on-chip case, *#delivery* is the number of time the MTP reacting chamber was refilled. In the off-chip case, this number corresponds to the increment of MTP reacting chamber volume that would be needed to treat the same surface of sample but using the standard manual protocol. The difference of reacting chambers height is hence taken into consideration. *Amount* is a subjectively formulated quantitative measure of the amount of reagents used. In essence it is number of molecule used divided by the volume of the MTP reacting chamber. The ratio is computed as on- to off-chip.
